# Renal Dysfunction after Off-Pump Coronary Artery Bypass Surgery- Risk Factors and Preventive Strategies

**Published:** 2009-08

**Authors:** Gaurab Maitra, Ahsan Ahmed, Amitava Rudra, Ravi Wankhede, Saikat Sengupta, Tanmoy Das

**Affiliations:** 1,4,5,6 Consultant, Department of Anesthesiology, Perioperative Medicine And Pain, Apollo Gleneagles Hospitals, Kolkata; 2 Resident, Department of Anesthesiology, Perioperative Medicine And Pain, Apollo Gleneagles Hospitals, Kolkata; 3 Hony Consultant, Department of Anesthesiology, Perioperative Medicine And Pain, Apollo Gleneagles Hospitals, Kolkata

**Keywords:** Off-pump CABG, Renal dysfunction, Risk factors, Preventive strategies

## Abstract

**Summary:**

Postoperative renal dysfunction is a relatively common and one of the serious complications of cardiac surgery. Though off-pump coronary artery bypass surgery technique avoids cardiopulmonary bypass circuit induced adverse effects on renal function, multiple other factors cause postoperative renal dysfunction in these groups of patients. Acute kidney injury is generally defined as an abrupt and sustained decrease in kidney function. There is no consensus on the amount of dysfunction that defines acute kidney injury, with more than 30 definitions in use in the literature today. Although serum creatinine is widely used as a marker for changes in glomerular filtration rate, the criteria used to define renal dysfunction and acute renal failure is highly variable. The variety of definitions used in clinical studies may be partly responsible for the large variations in the reported incidence. Indeed, the lack of a uniform definition for acute kidney injury is believed to be a major impediment to research in the field. To establish a uniform definition for acute kidney injury, the Acute Dialysis Quality Initiative formulated the Risk, Injury, Failure, Loss, and End-stage Kidney (RIFLE) classification. RIFLE, defines three grades of increasing severity of acute kidney injury – risk (class R), injury (class I) and failure (class F) – and two outcome classes (loss and end-stage kidney disease). Various perioperative risk factors for postoperative renal dysfunction and failure have been identified. Among the important preoperative factors are advanced age, reduced left ventricular function, emergency surgery, preoperative use of intraaortic balloon pump, elevated preoperative serum glucose and creatinine. Most important intraoperative risk factor is the intraoperative haemodynamic instability and all the causes of postoperative low output syndrome comprise the postoperative risk factors. The most important preventive strategies are the identification of the preoperative risk factors and therefore the high risk groups by developing clinical scoring systems. Preoperative treatment of congestive cardiac failure and volume depletion is mandatory. Avoidance of nephrotoxic drugs and prevention of significant hemodynamic events that may insult the kidney are essential. Perioperative hydration, aggressive control of serum glucose, haemodynamic monitoring and optimization of ventricular function are important strategies. Several drugs have been evaluated with inconsistent results. Dopamine and diuretics once thought to be renoprotective has not been shown to prevent renal failure. Mannitol is probably effective if given before the insult takes place. Some of the newer drugs like fenoldopam, atrial natriuretic peptide, N-acetylcysteine, clonidine and diltiazem have shown some promise in preventing renal dysfunction but more studies are needed to establish their role of renoprotection in cardiac surgery.

## Introduction

Postoperative renal dysfunction is are latively common and one of the serious complications of cardiac surgery. Renal dysfunction or failure occurs nearly in 8% of all patients undergoing myocardial revascularization^1^. It is multifactorial in origin^2^. Though off-pump coronary artery bypass surgery (OPCAB) technique avoids cardiopulmonary bypass (CPB) circuit induced adverse effects on renal function, multiple other factors cause postoperative renal dysfunction in these group of patients^3^‐^5^. Each year, 600,000 patients worldwide undergo coronary artery bypass surgery^1^. With an increasing number of elderly populations coming for coronary artery bypass surgery, clinicians will continually be challenged to mitigate peioperative renal failure. Compared with patients who do not have postoperative renal dysfunction, patients with renal dysfunction (who do not need dialysis) remain twice as long in both the intensive care unit and hospital wards and have significantly highermortality rate (1% compared with 19%)^1^,^6^,^7^. Furthermore, approximately 1 in 6 patients with renal dysfunction will need dialysis and two third of them will not survive their hospitalization^8^‐^11^. Many more patients suffer from occult, subclinical, and transient renal injury without requiring hemodialysis. Despite advances in surgical technique and better understanding of the pathophysiology of acute renal failure (ARF), mortality and morbidity associated with ARF have not markedly changed in the last decade^8^‐^16^. These data highlight the importance of identifying the risk factors associated with cardiac bypass surgery and implementing specific therapies that are based on the knowledge of well designed clinical trials. The lack of progress, though disappointing, offers an opportunity to ascertain why we have not been successful.

## Definitions and Incidence

Although serum creatinine (Cr) is widely used as a marker for changes in glomerular filtration rate (GFR), the criteria used to define renal dysfunction and acute renal failure (ARF) is highly variable-1‐4,12‐15. Depending upon the definition, the incidence varies across the studies. Some studies used the absolute serum Cr value alone and others used widely differing relative change criteria, this yielded incidence of renal dysfunction ranging from 3% to 29%^4^,^6^,^8^,^12^‐^15^. To establish a uniform definition for acute kidney injury, the Acute Dialysis Quality Initiative formulated the Risk, Injury, Failure, Loss, and End-stage Kidney (RIFLE) classification. RIFLE defines three grades of increasing severity of acute kidney injury – risk(class R), injury (class I) and failure (class F) – and two outcome classes (loss and end-stage kidney disease). A unique feature of the RIFLE classification is that it provides three grades of severity for acute kidney injury based on changes in either serum creatinine or urine output from the baseline condition. This allows classification of patients with acute kidney injury into one of the three RIFLE severity. Until recently, no consensus existed about how to best define, characterize, and study acute renal failure. This lack of a standard definition has been a major impediment to the progress of clinical and basic research in this field.

When using RIFLE criteria for assessment of renal dysfunction, two large retrospective studies indicate the incidence of acute kidney injury (AKI) after cardiac surgery is about 15% to 20%^17^,^18^. Most of the authors definerenal failure by the need for dialysis after surgery. Though serum Cr is used as a marker for GFR, it is seen that, GFR may be amore accurate parameter than serum Cr to predict long-term outcome^7^,^19^.

## Perioperative risk factors associated with ARF

Several studies have examined the risk factors associated with the development of postoperative renal failure. Most patients at increased risk for postoperative renal dysfunction can be identified before their surgical procedures. Certain preoperative risk factors have been repeatedly associated with an increased risk of ARF^1^,^19^‐^23^. These include female gender, advanced age, reduced left ventricular function or presence of congestive heart failure, diabetes mellitus, chronic obstructive pulmonary disease, peripheral vascular disease, need for emergent surgery, preoperative use of intraaortic balloon pump (IABP), elevated preoperative serum glucose, elevated preoperative serum creatinine or patients with preexisting renal disease. The incidence of postoperative ARF approximately doubles with one preoperative risk factor and quadruples with two risk factors^1^. Increased cardiopulmonary bypass or aortic cross-clamp time (>2hrs) have been considered intraoperative risk factors for postoperative renal dysfunction^1^. OPCAB (off-pump coronary artery bypass) obviously removes the intraoperative risk factors associated with cardiopulmonary bypass circuit. But the greater hemodynamic instability secondary to ventricular compression when the heart is manipulated in different positions to access the coronary arteries may be an important contributing factor in development of postoperative renal failure. Though one study^24^ showed that the choice of operative techniques (OPCAB vs on-pump CABG) was not associated with reduced renal morbidity, the bulk of data supported lower risk of ARF in patients who underwent OPCAB^25^‐^28^. Postoperative risk factors that critically affect renal function are those that cause or are markers of low output syndrome - myocardial infarction, hemorrhage, patients requiring IABP, patients with moderate to severe compromise in ventricular function congestive cardiac failure, or use of at least three inotropic drugs.^1^ Low cardiac output syndrome in the postoperative period after OPCAB is a high risk for developing ARF as the vulnerable kidney is subjected to marginal perfusion pressures. Patients requiring an IABP had a nearly sevenfold increase for postoperative renal dysfunction. Use of at least three inotropic drugs is associated with an increased risk for postoperative renal failure^1^.

## Pathogenesis

The pathologic changes in the kidney of patients with ARF following OPCAB are largely assumed to be due to acute tubular necrosis which is usually confirmed by granular casts in the urine. Hypoxia-ischemia is the predominant cause of perioperative ARF and results from low renal blood flow due to a reduced cardiac output; from regional factors reducing renal blood flow; or from disturbances of intrarenal blood flow related to inflammation, sepsis or toxin^29^. It was demonstrated that the transmembrane gradient for glomerular ultrafiltration was significantly diminished and there is a back-leak of glomerular ultrafiltrate across the injured epithelium. ARF begins with an early phase of vasomotor nephropathy in which there is associated alterations in vasoreactivity and renal perfusion leading to prerenalazotaemia and eventually cellular ATP depletion. These ultimately lead to mitochondrial dysfunction and accumulation of intracellular sodium, calcium and reactive oxygen species. Subsequently, multiple enzyme systems are activated and cause disruption of the cytoskeleton, membrane damage, nucleicacid degradation and cell death. Vascular endothelial cell injury induces vascular congestion, edema and infiltration of inflammatory cells. Furthermore, elaborations of inflammatory mediators lead to additional cellular injury^30^‐^35^.

## Preventive strategies

### Perioperative optimization of renal function

Prevention of postoperative renal dysfunction after OPCAB needs knowledge of identifying the preoperative risk factors. Several groups have developed clinical scoring systems that help to predict the risk^20^‐^23^,^36^. The aim is to select patients who are at risk and then to adopt strategies that would offer renal protection. Congestive cardiac failure and volume depletion should be treated preoperatively so as to increase cardiac output and therefore renal perfusion. Medications such as nonsteroidal antiinflammatory drugs (NSAIDs) and other nephrotoxic agents should be discontinued. Preventing significant hemodynamic events which may insult the kidney and meticulous postoperative care including optimizing ventricular function, aggressive control of serum glucose and close monitoring of fluid and renal status, perioperative hydration and use of hemodynamic monitoring and inotropic agents to optimize cardiac output are of important strategies^1^.

## Pharmacologic Interventions

Several drugs have been tried in attempting to reduce postoperative renal dysfunction with inconsistent results. Loop diuretics increase renal cortical blood flow^37^. However, several studies have shown no benefit and possibly even harm from perioperative diuretic therapy in cardiac surgical patients^37^‐^40^. Therefore, there is insufficient evidence to support the routine use of loop diuretics as specific renoprotective agents. Mannitol, an osmotic diuretic, has been evaluated in several studies of cardiac surgical patients^41^,^42^. In addition to the lack of beneficial effect on the kidney, studies have identified a nephrotoxic potential of high dose of mannitol especially in patients with preexisting renal insufficiency^43^. Nevertheless, it is probably effective in decreasingthe severity of the decline in GFR if given before the insult takes place but once the damage is established there is no evidence of therapeutic benefit^44^. Dopamine at low doses certainly interacts with vascular do paminergic receptors and stimulates diuresis and natriuresis^45^. However, use in high risk patients has failed to show benefits^46^ and it may have widespread adverse effects^47^. So, while dopamine and diuretics were once thought to be renoprotective, neither has demonstrated efficacy to prevent renal failure^48^. Fenoldopam, a selective D1 receptor antagonist, has shown some promise in the prevention of contrast-induced nephropathy^49^,^50^ though randomized controlled studies are very few evaluating the efficacy in postoperative renal dysfunction after cardiac surgery. Few studies showed reduction of renal dysfunction in patients after cardiac surgery^51^‐^53^, while other studies^54^,^55^ failed to show any renoprotective effect of fenoldopam. Therefore, more studies are needed to establish its role for renoprotection in cardiac surgery. Atrial Natriuretic Peptide (ANP) increases natriures is by increasing GFR as well as by inhibiting sodium reabsorption by the medullary collecting duct^56^. In a multicentric trial, anaritide, a 25-amino acid synthetic form of ANP was administered to critically ill patients to treat acute tubular necrosis. Whether patients received anaritide or not, dialysis free survival was the same for both the groups^57^. In other study, recombinant human ANP (rh ANP) was used to treat ARF after cardiac surgery with a significant reduction in the incidence of dialysis at day 21 after the start of the treatment^58^. N-acetylcysteine (N-AC) has been shown to block oxidant stress on cardiac surgery patients^59^ and may hold promise as a protective measure. Although it has been used in the prevention of contrast-induced nephropathy, two metaanalysis concluded that research on N-acetylcysteine and the incidence of contrast-induced nephropathy are too inconsistent to warrant any definitive conclusion on its efficacy^60^,^61^. Studies of N-AC to prevent postoperative dysfunction following cardiac surgery did not show any benefit^62^,^63^. In a recent study of N-AC to prevent acute kidney injury in cardiac surgery patients with pre-existing moderate renal insufficiency, N-AC did not cause a statistically significant improvement in postoperative estimated GFR; nonetheless its treatment effect was consistent with a plausible small to moderate benefit^64^. Therefore, N-AC should definitely be evaluated in large randomized trial. Activation of sympathetic system during and after cardiac surgery may lead to impairment of renal function. Two clinical trials using clonidine (an alpha2 agonist) has been used to attenuate these effects and have shown some promise in preventing deterioration of renal function after cardiac surgery^65^,^66^. The calcium channel blocker diltiazem has been evaluated as a renoprotective agent in cardiac surgery due to its renal vasodialatory effects^67^. In one study, perioperative infusion of diltiazem for 36 hrs has been shown to increase GFR significantly though tubular function was not influenced^68^. Another study with 24 hrs diltiazem infusion has shown no differences in postoperative serum creatinine levels^69^. Hyperglycemia is common in cardiac surgery and increased serum glucose in pre or intraoperative period is independently known to cause ARF after cardiac surgery^1^. Though there is no study of insulin as renoprotective agent for human cardiac surgery, ischemia-reperfusion injury models in rats showed significant benefit of insulin if used before the renal insult occured^70^. Prophylactic hemodialys is has also been attempted in a study in patients with highest risk for acute kidney injury which showed a reduction in postoperative ARF requiring dialysis than in the control group^71^. Still more randomized trials are needed to support the invasive approach before it can be broadly recommended.

The development of renal dysfunction after cardiac surgery is an independent predictor of poor outcome. We must develop a standard definition of ARF that is sensitive and specific to determine the true incidence of this complication, permit an accurate assessment of ARF on outcomes, and allow comparison of patients across centers. Early preventive measures may be a way of reducing postoperative ARE. Thus, sensitive markers of renal injury are desirable for early intervention to diminish and minimize the perioperative renal insults. Some recent studies demonstrate that sensitive markers of tubular injury may be altered much earlier than a rise in serum creatinine and may allow us to define the time points when injury occurs^72^,^73^. However, the studies on kidney-specific proteins vary widely with regard to the marker used, the study period, and the kind of patients and because different ‘gold standards’ of kidney dysfunction were used, neither a systematic review nor a meta-analysis is possible at present^74^. Therefore, future studies should be designed to identify high-risk individuals based on a score and provide timely interventions for prevention or amelioration of renal injury to obtain optimal outcomes.
